# Effects of Swedish Massage Therapy on Blood Pressure, Heart Rate, and Inflammatory Markers in Hypertensive Women

**DOI:** 10.1155/2013/171852

**Published:** 2013-08-18

**Authors:** Izreen Supa'at, Zaiton Zakaria, Oteh Maskon, Amilia Aminuddin, Nor Anita Megat Mohd Nordin

**Affiliations:** ^1^Faculty of Biomedical and Health Sciences, Universiti Selangor, Shah Alam, Malaysia; ^2^Physiology Department, Universiti Kebangsaan Malaysia Medical Centre, Kuala Lumpur, Malaysia; ^3^Medical Department, Cardiology Unit, Universiti Kebangsaan Malaysia Medical Centre, Kuala Lumpur, Malaysia

## Abstract

Swedish Massage Therapy (SMT) is known for its therapeutic relaxation effects. Hypertension is associated with stress and elevated endothelial inflammatory markers. This randomized control trial measured the effects of whole body SMT (massage group) or resting (control group) an hour weekly for four weeks on hypertensive women. Blood pressure (BP) and heart rate (HR) were measured before and after each intervention and endothelial inflammatory markers: vascular endothelial adhesion molecules 1 (VCAM-1) and intracellular adhesion molecules 1 (ICAM-1) were measured at baseline and after the last intervention. Massage group (*n=8*) showed significant systolic BP (SBP) reduction of 12 mmHg (*P=0.01*) and diastolic BP (DBP) reduction of 5 mmHg (*P=0.01*) after four sessions with no significant difference between groups. Reductions in HR were also seen in massage group after sessions 1, 3, and 4 with significant difference between groups. VCAM-1 showed significant reduction after four sessions: the massage group showed reduction of 998.05 ng/mL (*P=0.03*) and the control group of 375.70 ng/mL (*P=0.01*) with no significant differences between groups. There were no changes in ICAM-1. In conclusion, SMT or resting an hour weekly has effects on reducing BP, HR, and VCAM-1 in hypertensive women.

## 1. Introduction

The prevalence of hypertension in Malaysia is increasing from 32.9% in 1996 to 40.5% in 2004 for individuals above 30 years old [1] with a prevalence in women higher than men [2]. In 2004, 43% of women over 30 years old are hypertensive [3]. Hypertension is a major risk factor for cardiovascular disease (CVD). CVD is the primary cause of death in women in Malaysia as well as globally [4]. In addition, more women experienced the side effects of antihypertensive treatment such as calcium channel blockers and angiotensin converting enzyme inhibitors compared to men [4]. 

The development of primary hypertension has been closely associated with endothelial dysfunction. This was seen in the increased expression of interleukin 6 (IL-6), interleukin 1 (IL-1), tumour necrosis factor *α* (TNF-*α*), monocyte chemoattractant protein 1 (MCP-1), VCAM-1, and ICAM-1 in hypertensive rats [5–7]. VCAM-1 and ICAM-1 are immunoglobulin superfamily (IGSF) molecules involved in cell to cell adhesion. VCAM-1 is expressed on endothelial, epithelial, macrophage, and dendritic cells. ICAM-1 is expressed on endothelial, epithelial, fibroblast, leucocytes, and tumour cells. VCAM-1 and ICAM-1 form attachments and assist transendothelial migration of leukocytes at sites of atherosclerosis [8]. These molecules are upregulated in response to inflammatory cytokines or oxidative low-density lipoprotein (ox-LDL) [9]. During an inflammation caused by injury, leukocytes will roll on the endothelium before firmly adhering to the vessel wall. Stable adhesion of leukocytes further upregulates adhesion molecules that will drive the transmigration of leukocytes across the endothelium to the site of injury. Leukocytes adhesion is opposed by tangential tractive forces or shear stress induced by the blood flow velocity gradient near the vessel wall [10]. 

Shear stress is defined as the tangential drag force of blood passing along the surface of the endothelium [11]. Shear stress is directly proportional to blood viscosity and velocity and inversely proportional to blood vessel diameter (shear stress = blood viscosity × blood velocity/vessel diameter). There are two types of shear stress: the atheroprotective laminar or pulsatile shear stress which occurs in straight blood vessels [12] and the atherogenic oscillatory shear stress which occurs at bends and bifurcation of arteries [13–15]. The physiological atheroprotective shear stress is more than 15 dyne/cm^2^. In the event of narrowing of the arteries, the shear stress here is low. Consequently, blood will rush out of the narrow opening at high velocity and create a higher shear stress on the arterial wall, therefore inducing endothelial-dependent nitric oxide-mediated vasodilation which brings the shear stress back to normal. However, this effect is blunted in hypertensive and hypercholesterolemic patients [16]. It was found that with increasing shear stress the expression of VCAM-1 is low and the expression of ICAM-1 is high. There was also no leukocytes adhesion to the vessel wall. In contrast, if there is a decrease in shear stress, the expression of VCAM-1 increases and the expression of ICAM-1 decreases with leukocytes adhesion to the vessel wall. This is indicative of the early stage of development of atherosclerosis [17, 18]. 

Swedish Massage Therapy (SMT) is a complementary treatment that is believed to provide relaxation and therefore able to reduce blood pressure caused by stress [19]. It is the most recognised and frequent used massage therapy [20]. It is characterised by long strokes applied according to the venous and lymphatic flow. It is a painless, gentle and nonforceful technique that is not associated with any serious adverse effects [21]. Massage therapy has been shown to decrease sympathetic activity and increase parasympathetic activity [22]. Therefore this therapy is able to decrease anxiety and stress [23, 24]. In addition, massage therapy is able to reduce blood pressure (BP) and heart rate (HR) in hypertensive individuals [25–27]. It also increases skin blood flow and suppleness and induces tissue relaxation [28]. The long strokes in massage compress the body tissues and when released increase blood flow to the local area [19]. 

The present study assessed the effects of SMT versus rest on hypertensive women. If SMT yields positive results in this study, it can be recommended as an adjunct or a complementary therapy to the conventional management of hypertension especially in women as the prevalence of hypertension in women is high [1, 2]. As far as the author's present knowledge extends, no studies have assessed the effects of whole body Swedish Massage Therapy on hypertensive women on BP, HR, and inflammatory markers. In view of the literature stated above, it was hypothesized that massage increases blood flow and thus increases shear stress on the blood vessel wall. The increase in shear stress reduces the expression of VCAM-1 and vWF and increases ICAM-1. In addition, through the activation of parasympathetic nervous system, massage is able to reduce BP and HR. Therefore Swedish Massage Therapy is expected to reduce BP, HR, and VCAM-1 and increase ICAM-1. 

## 2. Methods

### 2.1. Participants

This study is an experimental randomized control trial that has been approved by the Ethics Committee of the Universiti Kebangsaan Malaysia Medical Centre (UKMMC) (Project Ethics Code: FF-280-2009). The participants were 35–60-year-old women recruited from the UKMMC records. These women must fulfil the following criteria:

- body mass index (BMI) of less than 35 kg/m2;
- SBP of 120–159 mmHg and DBP of 90–99 mmHg with or without treatment. If they are on antihypertensive or anticholesterol medications, they must be on only one medication of the same type and dose for at least six months;
- normal liver, thyroid, and renal functions;
- not taking any prescribed and/or traditional medications (apart from those stated in (b)) and supplements;
- not smoking or drinking alcohol;
- no other illnesses;
- not pregnant;
- have not experienced Swedish Massage Therapy.

Detailed explanation of the study was given to each woman. All women had signed the consent form before participating in this study. 

The sample size is calculated based on paired samples that is, BP taken before and after intervention with continuous outcomes. The formula used in this study is taken from Chan 2003 [29]. The values for this formula are taken from another study [26] with a similar topic and significant results. The minimum number of subjects calculated is 16 with 8 subjects per group.

Twenty-three women fulfilled the above criteria and were screened for any health conditions that may influence their blood pressure. Blood pressure of these women was monitored for two weeks prior to the intervention. Blood samples were taken to ensure that the liver, thyroid, and renal function and the fasting blood glucose level were normal. Resting electrocardiogram (ECG) and stress tests were also carried out to ensure normal cardiac function. Fasting body composition was measured before and after the intervention to ensure that the body fluid distribution remains unchanged. Twenty women successfully passed their screening. These women were randomly assigned to two groups: the massage group and the control group using random numbers generated through the SPSS version 15 software. However, only 16 women (8 per group) successfully completed the intervention.

### 2.2. Intervention

In this study, the massage protocol is an hour of Swedish Massage Therapy to the whole body, once a week for four weeks. An hour of massage allows enough time to apply all the Swedish massage techniques to the whole body which was expected to produce positive effects on BP and HR [30, 31]. Massage sessions once a week for four weeks are considered not too frequent as they prevent the subject from showing any signs of relaxation prior to the massage session as would be expected if the sessions are more frequent. 

Eight women in the massage group underwent an hour of whole body Swedish Massage Therapy once a week for four weeks at the Clinical Trial Ward, UKMMC. A qualified massage therapist with a certificate in Holistic Therapy from the Institute of Bioproduct Development, Universiti Teknologi Malaysia, carried out the massage on each of the subjects. The massage techniques used are a combination of *petrissage *or kneading, *tapotement* or beating/hacking/cupping, and* effleurage *or long strokes. These techniques are applied at medium pressure. Olive oil was used as the lubricant. These massage sessions were carried out during working days between 8 and 10 a.m. The protocol used is described below.

1. The subject is requested to lie prone with only the right leg is exposed. Massage oil applied on the exposed leg.
2. Long strokes are applied on the posterior right leg.
3. The gastrocnemius muscle is kneaded using both thumbs.
4. Step (2) is repeated.
5. The medial and lateral parts of back of the thigh are
  - kneaded using the palm of the hand;
  - hacked or striken with the medial side of the hand;
  - pounded using the medial side of a clenched fist.
6. Lymphatic drainage is then carried out by applying long strokes along the venous or lymphatic vessels towards the nearest lymph nodes.
7. Step (2) is repeated.
8. The left leg is covered and steps 1–7 are repeated on the left leg.
9. Massage is then carried out on the back. Massage oil is applied on the whole back.
10. Long strokes are carried out using the palms of therapist hands from the lower back to the shoulders.
11. Kneading using fingers is applied parallel to the spine from the lower back to between the scapulae.
12. Step (10) is repeated.
13. Kneading using palms is applied on both loin areas and posterior to the lungs.
14. Lymphatic drainage was carried out from the lower back to the axillary and subclavicular lymph nodes.
15. Step (10) is repeated.
16. The subject is requested to turn over and lie supine with the right leg exposed. Massage oil is applied on the whole leg.
17. Long strokes are applied on the anterior right leg.
18. The anterior tibialis muscle is kneaded using both thumbs.
19. Step (17) is repeated.
20. The biceps femoris is kneaded using the palm of the hand.
21. Step (6) and Step (17) are repeated.
22. Massage is then carried out on the abdomen where massage oil is applied.
23. Long strokes are applied from the umbilicus to the xiphisternum, along the lower border of the ribs towards lateral of the abdomen and inferior towards the inguinal area.
24. Strokes are applied along the ascending, transverse, and descending colon.
25. Small, circular kneading using tips of fingers is applied clockwise around the umbilicus.
26. Lymphatic drainage is carried out through long strokes from the posterior loin area to the inguinal region.
27. Step (23) is repeated.
28. Massage is then carried out on the right arm where massage oil is applied.
29. Effleurage is applied using one hand to support the subject's arm and the other hand carrying out long strokes from the wrist to the scapula region.
30. The forearm is kneaded using the thumb.
31. The upper arm is kneaded using the palm of the hand.
32. Step (29) is repeated.
33. The subject is requested to sit up with her back to the massage therapist.
34. The massage therapy ends with massage to the scalp, neck, and shoulders.
35. The scalp is kneaded from the frontal area to the occipital area using fingers.
36. The temples are kneaded in circular motions using the heel of the hands.
37. The trapezius and deltoid muscles are kneaded according to the orientation of the muscle fibers using fingers.
38. BP and HR were taken. The subject is requested to dress.

Eight women in the control group underwent an hour of rest, once a week for four weeks at the same ward and at the same time. They were instructed to lie supine and rest either doing light reading or sleep. They are not allowed to listen to any music, watch television, exercise, or carry out any activities that may affect their BP. 

### 2.3. Measurements

Blood pressure and HR were taken before and after each massage and rest session and 48 hours after the last session. Blood pressure was taken twice at five-minute interval using the mercury sphygmomanometer and HR through palpation. 

10 mL of blood was collected in normal test tubes with no chemical preservatives at baseline, that is, before the first massage and rest session and after the last session ended. The blood was left to coagulate for two hours before being centrifuged at 3000 rpm for 10 minutes at 4°C. The serum is pipetted into 1 mL Eppendorf tubes and stored at −70°C before being analysed. The level of soluble VCAM-1 and ICAM-1 was analysed using kits through the enzyme-linked immunosorbent assay method (ELISA). The analysis protocols were carried out as instructed in the kits: VCAM-1 (BMS232TEN, Bender MedSystems, Austria) and ICAM-1 (BMS201INST, Bender MedSystems, Austria). The concentration of soluble VCAM-1 and ICAM-1 of each sample was calculated through the SOFTmaxPRO software through the measurements of the absorbance value of the study samples, the standard samples, and the control samples. The normal range of soluble VCAM-1 as stated in the kit is 400.6–1340.8 ng/mL and soluble ICAM-1 is 129.9–297.4 ng/mL. 

### 2.4. Statistical Analyses

Data were analysed using SPSS software version 15. Due to the small number of samples (<20), nonparametric statistical tests were used. The Mann-Whitney *U* test was used to compare the readings between the two, groups, and the Wilcoxon Signed Rank test was used to measure the changes in each group. For BP and HR, the difference between the preintervention readings and postintervention readings of each session is considered to be an acute change. The difference between the preintervention readings of BP and HR of session 1 and preintervention readings of subsequent sessions is considered to be chronic change. A four week chronic change is the difference between the preintervention reading of BP and HR of session 1 and reading 48 hours after session 4. All data is presented in median (interquartile range). The value of *P* < 0.05 is accepted as the significant level. 

## 3. Results

### 3.1. Baseline Characteristics

The baseline characteristics of the research participants are shown in Table 1. Women in the massage and control groups were comparable in all parameters except for a higher fasting blood glucose level in the latter. However, this was still within the normal range. In general, these women were overweight; resting BP showed Stage 1 hypertension, and lipid profile showed high cholesterol and high LDL while HDL levels were normal. More than 62% were nonmenopausal. In each group, three participants (37.5%) are on antihypertensive medications and only one (12.5%) is on anticholesterol medication. 

### 3.2. SBP Changes

**Figure 1:**
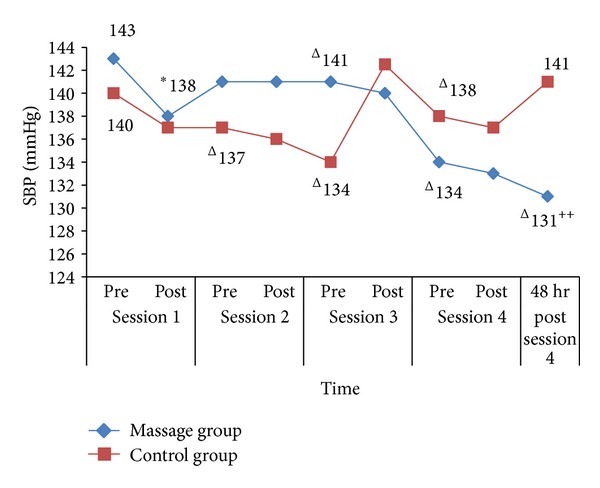
SBP changes for each session for massage group and control group. *P < 0.05 (acute changes within groups) and ^∆^ *P* < 0.05 (chronic changes within groups). ^++^ *P* < 0.05 (baseline versus after session 4).

Figure 1 shows the trend of SBP changes for every session for both groups. During session 1, there was an acute significant reduction in the massage group from 143.00 (9.00) mmHg to 138.00 (16.75) mmHg (*Z* = −1.70, *P* = 0.03). Chronic significant reduction was seen after the first week in the control group from 140.00 (23.75) mmHg to 137.00 (11.75) mmHg with *Z* = −2.52, *P* = 0.01, after the second and third weeks in both groups and after the fourth week in the massage group only at 12 mmHg (*Z* = −2.54, *P* = 0.01). There was no significant difference in SBP readings between massage and control groups. 

### 3.3. DBP Changes

**Figure 2:**
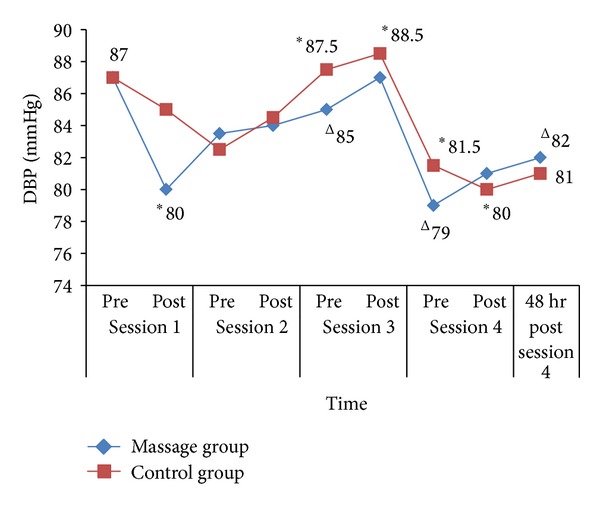
DBP changes for each session for massage group and control group. *P < 0.05 (acute changes within groups) and ^∆^ *P* < 0.05 (chronic changes within groups).

The trend in DBP changes is shown in Figure 2. During session 1, there was an acute significant DBP reduction of 7 mmHg in massage group (*Z* = −2.38, *P* = 0.02). During session 4, there was an acute significant reduction in the control group from 81.50 (8.75) mmHg to 80.00 (5.25) mmHg with *Z* = −2.03, *P* = 0.04. In addition, chronic significant DBP reductions were seen after the second week (−2 mmHg, (*Z* = −2.21, *P* = 0.03)), the third week (−8 mmHg, (*Z* = −2.54, *P* = 0.01)), and the fourth week (−5 mmHg, (*Z* = −2.52, *P* = 0.01)) in the massage group. There were no significant differences between the two groups in all DBP readings. 

**Figure 3:**
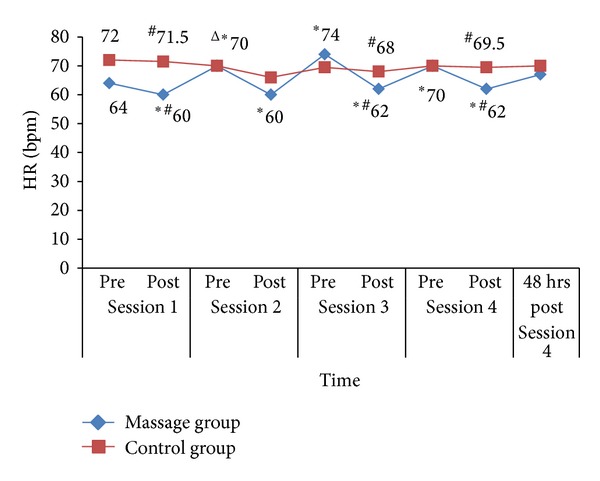
HR changes for each session for massage group and control group. *P < 0.05 (acute changes within groups), ^∆^ *P* < 0.05 (chronic changes within groups), and ^#^ *P* < 0.05 (changes between groups).

Figure 3 shows the trend of HR changes. There is an acute significant HR reduction in massage group after each session with significant differences between groups after session 1 (*Z* = −2.22, *P* = 0.03), session 3 (*Z* = −2.07, *P* = 0.04), and session 4 (*Z* = −2.03, *P* = 0.04). Chronic reduction was seen in the control group after the first week (*Z* = −2.26, *P* = 0.02) with no significant difference between groups. 

### 3.4. VCAM-1 Changes

**Figure 4:**
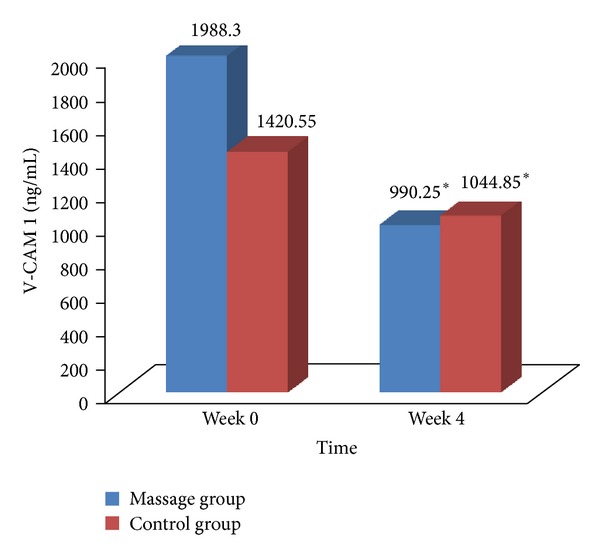
VCAM-1 changes for massage group and control group. *P < 0.05 (reduction within groups).

The changes in the level of VCAM-1 are displayed in Figure 4. Significant reduction in VCAM-1 was seen in massage group from 1988.30 (911.39) ng/mL to 990.25 (675.25) ng/mL (*Z* = − 2.20, *P* = 0.03) and the control group from 1420.55 (861.07) ng/mL to 1044.85 (602.39) ng/mL (*Z* = − 2.52, *P* = 0.01). No significant change was seen between groups. 

### 3.5. ICAM-1 Changes

**Figure 5:**
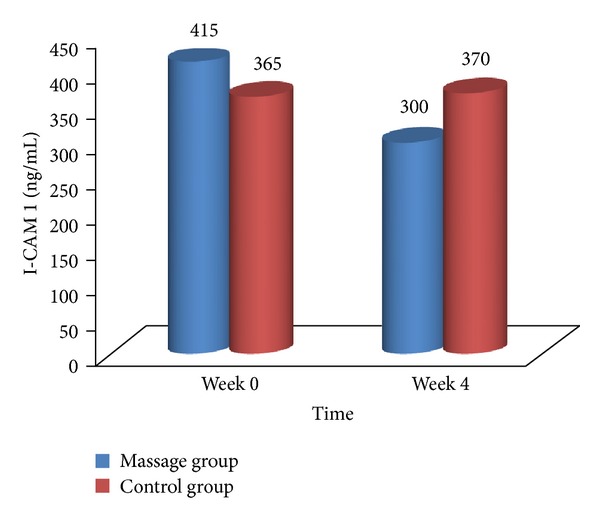
ICAM-1 changes for massage group and control group.

No significant changes were seen within the two groups and between groups as shown in Figure 5. 

## 4. Discussion

Massage group showed chronic significant reduction in SBP after two, three, and four weeks. These results are consistent with Olney (2005) [25], Hernandez-Reif et al. (2000) [26], and Moeini et al. (2011) [27] which showed that massage had long-term effects on the BP of hypertensive patients. However, the control group of this current study also showed chronic significant reduction in SBP after one, two, and three weeks. These results contradict the results of the authors mentioned. Olney (2005) [25], Hernandez-Reif et al. (2000) [26], and Moeini et al. (2011) [27] showed no significant changes in SBP of their control groups. Several factors may be able to explain these occurrences. Firstly, the rest session of this current study is of longer duration if compared to the studies of Olney [25] and Moeini et al. [27]. Secondly, during the resting period, the subject is free to use any relaxation methods, and the researcher is only present during the pre- and postintervention measurements. The combination of no supervision and longer duration of rest may have reduced the SBP of the control subjects. Acute reduction in SBP of 5 mmHg after session 1 in the massage group was also seen, and this is consistent with the results of Hernandez-Reif et al. (2000) [26].

In parallel with the results of SBP, the massage group also showed significant chronic reduction in DBP after two, three, and four weeks of massage therapy and acute significant reduction of 7 mmHg after session 1. In summary, SMT is able to reduce both SBP and DBP, even though there is no significant difference between groups. 

Heart rate of the massage group was reduced significantly after each session, and the changes were significant between groups after sessions one, three, and four. Even though the duration of the massage session is different from other studies, these results are consistent with studies that measure the effects of massage on normal individuals [22], breast cancer patients [32], hospice patients [33], migraine patients [34], and critical care patients [35]. 

The reduction in BP and HR could be explained through the comfortable feeling and relaxation, as well as the increase in parasympathetic activities induced by massage as shown by Ouchi et al. (2006) [22]. This is supported by Diego and Field (2009) [36] who showed that the massage applied at medium pressure for 15 minutes caused increase in the high-frequency component of HR variability which reflected an increase in vagal activities. In addition, there was a decrease in the ratio of low-frequency component to high-frequency component of HR variability which indicates a change from sympathetic activities to parasympathetic activities. 

To date, there have been no studies that measure the effects of massage on endothelial inflammatory markers. In this current study, the massage group experienced a significant reduction of VCAM-1 from an abnormal level to a normal level with a greater magnitude difference compared to the control group which also showed significant reduction. Braun and Simonson 2008 [20] stated that SMT through *effleurage* and compression increases local blood flow. If blood viscosity remains unchanged, the increase in blood flow increases shear stress on the blood vessel wall. The increase in shear stress decreases the expression of VCAM-1 [18, 37]. This is supported by the studies of Ando et al., (1994) [17], Korenaga et al. (1997)[38], and Helmlinger et al. (1995)[39] which showed decrease in the production of VCAM-1 at physiological shear stress of >15 dyne/cm^2^ and an increase in the production of VCAM-1 at shear stress of ±0–4 dyne/cm^2^.

It was expected that the level of ICAM-1 of the massage group increases after the intervention. Walpola et al. (1995) [18] showed that high shear stress (30.5 dyne/cm^2^) increases the expression of ICAM-1. Nagel et al. (1994) [40] who studied the effects of shear stress at 10 dyne/cm^2^ on the expression of ICAM-1 on human umbilical vein endothelial cells (HUVEC) and Morigi et al. (1995) [10] who exposed HUVEC at shear stress of 8 dyne/cm^2^ also reported an increase in the expression of ICAM-1. However, the current study showed no significant changes in both massage and control groups for ICAM-1. It may be that the shear stress created by massage is not large enough to have effects on ICAM-1. Further studies are warranted on the effects of massage on blood flow to confirm the effects discussed above. 

## 5. Conclusion

This study has shown that Swedish Massage Therapy or resting an hour weekly significantly reduced BP, HR, and VCAM-1 through the effects that have been discussed. However, the effect of rest on BP does not extend to four weeks as compared to SMT. In addition, massage also reduces resting HR in hypertensive women.

## Figures and Tables

**Table 1:** Baseline characteristics of the research participants.

Parameters	Massage group (*n* = 8)	Control group (*n* = 8)	*P*
Age (years)	51.00 (10.00)	51.13 (11.00)	0.96
Employed (%)	50	37.5	0.61
Nonmenopausal (%)	62.5	75	0.59
On antihypertensives (%)	37.5	37.5	1.00
On anticholesterol (%)	12.5	12.5	1.00
Resting SBP (mmHg)	142.25 (18.38)	143.75 (7.50)	0.67
Resting DBP (mmHg)	81.25 (9.75)	89.50 (13.50)	0.53
Resting HR (bpm)	66.00 (15.50)	72.00 (3.00)	0.10
BMI (kg/m^2^)	29.02 (6.65)	28.15 (7.41)	0.83
Cholesterol (mmol/L)	5.80 (0.74)	6.42 (1.32)	0.13
HDL (mmol/L)	1.30 (0.42)	1.41 (0.20)	0.11
LDL (mmol/L)	3.95 (0.32)	4.44 (1.68)	0.49
Fasting blood glucose (mmol/L)*	4.95 (0.25)	5.50 (0.45)	0.01

Data in median (interquartile range). *P < 0.01 is significant.

## References

[1] Rampal L, Rampal S, Azhar MZ, Rahman AR (2008). Prevalence, awareness, treatment and control of hypertension in Malaysia: a national study of 16,440 subjects. *Public Health*.

[2] Lim TO, Morad Z, Hussein RH (2004). Prevalence, awareness, treatment and control of hypertension in the Malaysian adult population: results from the National Health and Morbidity Survey 1996. *Singapore Medical Journal*.

[4] MOH/P/PAK/171.08(GU) (2008). *Clinical Practice Guidelines: Prevention of Cardiovascular Disease in Women*.

[5] Sanz-Rosa D, Oubiña MP, Cediel E (2005). Effect of AT1 receptor antagonism on vascular and circulating inflammatory mediators in SHR: role of NF-*κ*B/I*κ*B system. *American Journal of Physiology*.

[6] Capers Q, Alexander RW, Lou P (1997). Monocyte chemoattractant protein-1 expression in aortic tissues of hypertensive rats. *Hypertension*.

[7] Luvarà G, Pueyo ME, Philippe M (1998). Chronic blockade of NO synthase activity induces a proinflammatory phenotype in the arterial wall: prevention by angiotensin II antagonism. *Arteriosclerosis, Thrombosis, and Vascular Biology*.

[8] Gearing AJH, Newman W (1993). Circulating adhesion molecules in disease. *Immunology Today*.

[9] Constans J, Conri C (2006). Circulating markers of endothelial function in cardiovascular disease. *Clinica Chimica Acta*.

[10] Morigi M, Zoja C, Figliuzzi M (1995). Fluid shear stress modulates surface expression of adhesion molecules by endothelial cells. *Blood*.

[11] Gnasso A, Irace C, Carallo C (1997). In vivo association between low wall shear stress and plaque in subjects with asymmetrical carotid atherosclerosis. *Stroke*.

[12] Traub O, Berk BC (1998). Laminar shear stress: mechanisms by which endothelial cells transduce an atheroprotective force. *Arteriosclerosis, Thrombosis, and Vascular Biology*.

[13] Ku DN, Giddens DP, Zarins CK, Glagov S (1985). Pulsatile flow and atherosclerosis in the human carotid bifurcation. Positive correlation between plaque location and low and oscillating shear stress. *Arteriosclerosis*.

[14] Ravensbergen J, Ravensbergen JW, Krijger JKB, Hillen B, Hoogstraten HW (1998). Localizing role of hemodynamics in atherosclerosis in several human vertebrobasilar junction geometries. *Arteriosclerosis, Thrombosis, and Vascular Biology*.

[15] Zarins CK, Giddens DP, Bharadvaj BK (1983). Carotid bifurcation atherosclerosis. Quantitative correlation of plaque localization with flow velocity profiles and wall shear stress. *Circulation Research*.

[16] Paniagua OA, Bryant MB, Panza JA (2001). Role of endothelial nitric oxide in shear stress-induced vasodilation of human microvasculature: diminished activity in hypertensive and hypercholesterolemic patients. *Circulation*.

[17] Ando J, Tsuboi H, Korenaga R (1994). Shear stress inhibits adhesion of cultured mouse endothelial cells to lymphocytes by downregulating VCAM-1 expression. *American Journal of Physiology*.

[18] Walpola PL, Gotlieb AI, Cybulsky MI, Langille BL (1995). Expression of ICAM-1 and VCAM-1 and monocyte adherence in arteries exposed to altered shear stress. *Arteriosclerosis, Thrombosis, and Vascular Biology*.

[19] Fritz S (2009). *Mosby's Fundamentals of Therapeutic Massage*.

[20] Braun MB, Simonson SJ (2008). *Introduction to Massage Therapy*.

[21] Ernst E (2003). The safety of massage therapy. *Rheumatology*.

[22] Ouchi Y, Kanno T, Okada H (2006). Changes in cerebral blood flow under the prone condition with and without massage. *Neuroscience Letters*.

[23] Richards KC, Gibson R, Overton-McCoy AL (2000). Effects of massage in acute and critical care. *AACN Clinical Issues*.

[24] Moraska A, Pollini RA, Boulanger K, Brooks MZ, Teitlebaum L (2010). Physiological adjustments to stress measures following massage therapy: a review of the literature. *Evidence-Based Complementary and Alternative Medicine*.

[25] Olney CM (2005). The effect of therapeutic back massage in hypertensive persons: a preliminary study. *Biological Research for Nursing*.

[26] Hernandez-Reif M, Field T, Krasnegor J, Hossain Z, Theakston H, Burman I (2000). High blood pressure and associated symptoms were reduced by massage therapy. *Journal of Bodywork and Movement Therapies*.

[27] Moeini M, Givi M, Ghasempour Z, Sadeghi M (2011). The effect of massage therapy on blood pressure of women with pre-hypertension. *Iranian Journal of Nursing & Midwifery Research*.

[28] Duimel-Peeters IGP, Halfens RJG, Berger MPF, Snoeckx LHEH (2005). The effects of massage as a method to prevent pressure ulcers. A review of the literature. *Ostomy/Wound Management*.

[29] Chan YH (2003). Randomised controlled trials (RCTS)—sample size: the magic number?. *Singapore Medical Journal*.

[30] Lovas JM, Craig AR, Segal YD, Raison RL, Weston KM, Markus MR (2002). The effects of massage therapy on the human immune response in healthy adults. *Journal of Bodywork and Movement Therapies*.

[31] Aourell M, Skoog M, Carleson J (2005). Effects of Swedish massage on blood pressure. *Complementary Therapies in Clinical Practice*.

[32] Billhult A, Lindholm C, Gunnarsson R, Stener-Victorin E (2009). The effect of massage on immune function and stress in women with breast cancer—a randomized controlled trial. *Autonomic Neuroscience: Basic and Clinical*.

[33] Meek SS (1993). Effects of slow stroke back massage on relaxation in hospice clients. *Journal of Nursing Scholarship*.

[34] Lawler SP, Cameron LD (2006). A randomized, controlled trial of massage therapy as a treatment for migraine. *Annals of Behavioral Medicine*.

[35] Hayes JA, Cox C (2000). Immediate effects of a five-minute foot massage on patients in critical care. *Complementary Therapies in Nursing & Midwifery*.

[36] Diego MA, Field T (2009). Moderate pressure massage elicits a parasympathetic nervous system response. *International Journal of Neuroscience*.

[37] Walpola PL, Gotlieb AI, Langille BL (1993). Monocyte adhesion and changes in endothelial cell number, morphology, and F-actin distribution elicited by low shear stress in vivo. *American Journal of Pathology*.

[38] Korenaga E, Ando J, Kosaki K, Isshiki M, Takada Y, Kamiya A (1997). Negative transcriptional regulation of the VCAM-1 gene by fluid shear stress in murine endothelial cells. *American Journal of Physiology*.

[39] Helmlinger G, Berk BC, Nerem RM (1995). Calcium responses of endothelial cell monolayers subjected to pulsatile and steady laminar flow differ. *American Journal of Physiology*.

[40] Nagel T, Resnick N, Atkinson WJ, Dewey CF, Gimbrone MA (1994). Shear stress selectively upregulates intercellular adhesion molecule-1 expression in cultured human vascular endothelial cells. *Journal of Clinical Investigation*.

